# Errata

**DOI:** 10.36660/abc.20200954

**Published:** 2020-09-18

**Edição de Fevereiro de 2020, vol. 114 (2), págs. 305-312**

No Artigo de Revisão “Alterações Cardiovasculares em Pacientes Portadores de Lipodistrofia Familiar”, com número de DOI: https://doi.org/10.36660/abc.20190016, publicado no periódico Arquivos Brasileiros de Cardiologia, 114(2):305-312, na página 305, onde se lia:

Paula Ananda Inês Chacon

Leia-se:

Paula Ananda Chacon Inês

na página 311, onde lia:

Chacon PAI

Leia-se:

Inês PAC

**Edição de Agosto de 2020, vol. 115 (2), págs. 284-291**

No “Posicionamento do Departamento de Ergometria, Exercício, Cardiologia Nuclear e Reabilitação Cardiovascular (DERC/SBC) sobre a Atuação Médica em suas Áreas Durante a Pandemia por COVID-19”, com número de DOI: https://doi.org/10.36660/abc.20200797, publicado no periódico Arquivos Brasileiros de Cardiologia, 115(2): 284-291, na página 290, onde se lia:

“Qualquer que seja a atividade física regular escolhida, só deve ser reiniciada após negativação da PCR e liberação clínica.”

O correto é:

“Para pessoas previamente diagnosticadas com Covid-19 sintomático, que permanecem assintomáticos após a recuperação, um novo teste não é recomendado dentro dos próximos 3 meses após a data do início da infecção, e a liberação para atividade física dependerá da liberação clínica.”

