# Supplementary material for: Errata
Source: Arq Bras Cardiol. 2020 Sep 18;115(3):592. [Article in Portuguese] doi: 10.36660/abc.20200954 (PMC9363083; doi:10.36660/abc.20200954)
Supplement: Translation

# Translation: Erratum

**February 2020 Issue, vol. 114 (2), pages 305-312**

In the Review Article “Cardiac Alterations in Patients with Familial Lipodystrophy”, with DOI number: https://doi.org/10.36660/abc.20190016, published in the journal *Arquivos Brasileiros de Cardiologia,* 114(2):305-312, on page 305, where you read:

Paula Ananda Inês Chacon

Read:

Paula Ananda Chacon Inês

on page 311, where you read:

Chacon PAI

Read:

Inês PAC

**August 2020 Issue, vol. 115 (2), pages 284-291**

In the “Position Statement of the Brazilian Society of Cardiology Department of Exercise Testing, Sports Exercise, Nuclear Cardiology, and Cardiovascular Rehabilitation (DERC/SBC) on Activities Within its Scope of Practice During the COVID-19 Pandemic”, with DOI number: https://doi.org/10.36660/abc.20200797, published in the journal *Arquivos Brasileiros de Cardiologia,* 115(2):284-291, on page 290, where you read:

“Whichever regular physical activity is desired, clearance for practice is contingent upon negative PCR and clinical reassessment.”

The correct is:

“For people previously diagnosed with symptomatic Covid-19 who remain asymptomatic after recovery, a new test is not recommended within the next 3 months after the onset date of infection, and release for physical activity will depend on clinical evaluation.”
